# ShallowHRD: detection of homologous recombination deficiency from shallow whole genome sequencing

**DOI:** 10.1093/bioinformatics/btaa261

**Published:** 2020-04-21

**Authors:** Alexandre Eeckhoutte, Alexandre Houy, Elodie Manié, Manon Reverdy, Ivan Bièche, Elisabetta Marangoni, Oumou Goundiam, Anne Vincent-Salomon, Dominique Stoppa-Lyonnet, François-Clément Bidard, Marc-Henri Stern, Tatiana Popova

**Affiliations:** b1 DNA Repair and Uveal Melanoma (D.R.U.M.), Inserm U830, Institut Curie, Paris 75248, France; b2 Institut Curie, PSL Research University, Paris 75005, France; b3 Department of Genetics, Institut Curie, Paris 75248, France; b4 Department of Translational Research, Institut Curie PSL Research University, Paris 75248, France; b5 Department of Biopathology, Institut Curie PSL Research University, Paris 75005, France; b6 Faculty of Medicine, University of Paris, Paris, France; b7 Department of Medical Oncology, Institut Curie PSL Research University, Paris 75248, France; b8 Versailles Saint Quentin en Yvelines University, Paris Saclay University, Versailles 78035, France

## Abstract

**Summary:**

We introduce *shallowHRD*, a software tool to evaluate tumor homologous recombination deficiency (HRD) based on whole genome sequencing (WGS) at low coverage (shallow WGS or sWGS; ∼1X coverage). The tool, based on mining copy number alterations profile, implements a fast and straightforward procedure that shows 87.5% sensitivity and 90.5% specificity for HRD detection. *shallowHRD* could be instrumental in predicting response to poly(ADP-ribose) polymerase inhibitors, to which HRD tumors are selectively sensitive. *shallowHRD* displays efficiency comparable to most state-of-art approaches, is cost-effective, generates low-storable outputs and is also suitable for fixed-formalin paraffin embedded tissues.

**Availability and implementation:**

*shallowHRD* R script and documentation are available at https://github.com/aeeckhou/shallowHRD.

**Supplementary information:**

Supplementary data are available at *Bioinformatics* online.

## 1 Introduction

Aggressive subtypes of breast and ovarian cancers are frequently associated with homologous recombination deficiency (HRD) making these tumors sensitive to poly(ADP-ribose) polymerase inhibitors (Coleman *et al.*, 2019). HRD arises upon inactivation of *BRCA1/2*, *RAD51C* or *PALB2* and is characterized by specific tumor genome instability (Nik-Zainal *et al.*, 2016; Staaf *et al.*, 2019). Even though HRD genes are mostly known, exhaustive testing of their inactivation is difficult. This motivates developing surrogate genomic markers of HRD. Recent developments based on high throughput sequencing, HRDetect, Signature 3, SigMA, scarHRD, achieved excellent capacity to evaluate HRD (Davies *et al.*, 2017; Gulhan *et al.*, 2019; Polak *et al.*, 2017; Sztupinszki *et al.*, 2018). However, these methods are technically complex, time- and data-storage consuming, often need a matched normal sample and can be costly.

We introduce *shallowHRD*, a software for HRD testing based on the number of large-scale genomic alterations (LGA) obtained from whole genome sequencing (WGS) at low coverage (shallow WGS or sWGS; ∼1X). sWGS robustly detect copy number alterations (CNAs), even in fixed-formalin paraffin embedded (FFPE) samples and liquid biopsies (Van Roy *et al.*, 2017) at low cost and with easy-storable outputs. The concept of LGAs follows single-nucleotide polymorphism (SNP) array approaches, exploiting an increased number of large-scale intra-chromosomal CNAs characteristic of HRD (Abkevich *et al.*, 2012; Birkbak *et al.*, 2012; Popova *et al.*, 2012).

## 2 Materials and methods

### 2.1 Data

In-house sWGS of breast and ovarian cancers (26 primary tumors, 39 patient-derived xenografts from frozen blocks and 4 primary tumors FFPE) and down-sampled to ∼1X WGS (108 normal tissues, 79 primary tumors from the TCGA breast cancer) were processed by Control-FREEC (v11.5) (Boeva *et al.*, 2012) (Supplementary Material).

### 2.2 shallowHRD

The tool takes as input ‘*sample_name*.bam_ratio.txt’, which includes CNA profile ${\left\{x,\;g\right\}}_{1,\;N}$ where *x* is normalized read counts in a sliding window, g is genomic coordinate and the profile segmentation with *S_i_*, *Z_i_* segment median and size (in megabases, Mb).

#### 2.2.1 Workflow

- CNA cut-off is detected and the profile segmentation is optimized as follows: Segments are defined as ‘large’ if $Z_{i}\geq (Q1+Q3)/2$, where Q1, Q3 are quartiles of *Z_i_* (*Z_i_* > 3 Mb) distribution. *M* is detected as the first local minimum of $\left|\left(S_{i}-S_{j}\right)\right|$ density, where *i*, *j* are large segments (Supplementary Fig. S1). $\mathrm{CNA}\;\text{cut}-off=\;\min\left(\max\left(0.025,\;M\right),\;0.45\right)$. Adjacent segments are merged if $\left|\left(S_{i}-S_{i+1}\right)\right|<\text{CNA}\;\text{cut}-\text{off}$; starting from the largest segment.
- LGAs, defined as intra-chromosome arm CNA breaks with adjacent segments $Z_{i},Z_{i+1}\geq 10\;$Mb, are counted after removing segments <3 Mb.
- The sample is annotated as ‘non-HRD’ (LGA < 15), ‘borderline’ (15 ≤ LGA ≤ 19) or ‘HRD’ (LGA > 19).
- Sample quality is defined by *M* and *cMAD*, $\mathrm{cMAD}\;=\mathrm{median}\left(\left|\left(x-S_{x}\right)\right|\right)$, where $S_{x}$ corresponds to the segment enclosing *x*, before optimization: ‘bad’ (*cMAD* > 0.5 | *cMAD* > 0.14 and *M* > 0.45), ‘average’ (*cMAD* > 0.14 and *M* < 0.45 | *cMAD* < 0.14 and *M* > 0.45) or ‘normal or highly contaminated’ (*M* < 0.025) (Supplementary Material and Fig. S2).
- *CCNE1* amplification is called if $S_{c}\geq 4\cdot \text{CNA}\;\text{cut}-\text{off}$, where *c* is the segment enclosing the gene (4 was set arbitrarily).

*shallowHRD* output contains: (A) Tumor genome profile. (B) Density plot for CNA cut-off. (C) CNA segmentation summary. (D) Sample quality and HRD diagnostics (Supplementary Fig. S3).

## 3 Results

In-house sWGS and down-sampled WGS of normal samples (TCGA) were employed to develop the sWGS methodology similar to the large-scale state transitions (LST) in SNP-arrays (Popova *et al.*, 2012) (Section 2). LGAs inferred from sWGS corresponded well to the LSTs with identical HRD calls for 8 primary tumors tested (76–97% match in segments ≥ 10 Mb) (Supplementary Fig. S4). sWGS coverage >0.3X provide adequate quality, also for FFPE (Supplementary Figs. S2 and 5).

**Fig. 1.**
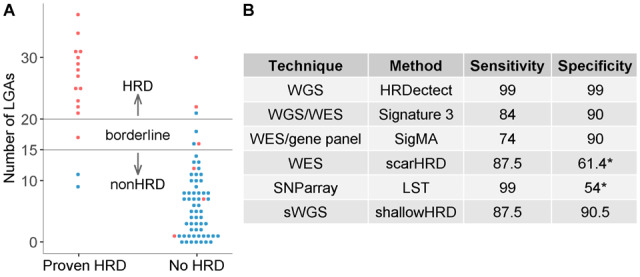
*shallowHRD* validation in down-sampled WGS of the TCGA (**A**) and performance (**B**). Proven/No HRD: cases with/without inactivation of *BRCA1/2*, *RAD51C* or *PALB2* (Supplementary Material); HRD (red) and non-HRD (blue) cases in SNP-arrays; LGAs: large-scale genomic alterations; WES: whole exome sequencing. ^a^Low specificity could be due to non-complete annotation of HRD

Validation by down-sampled WGS (TCGA) showed LGA to be coherent to SNP-arrays LST (*r* = 0.92; slope = 0.88; *P* < 2.2e–16, Pearson) with increased discrepancy in average quality samples (*n* = 13), and HRD diagnostics discordant in three and borderline in four cases (Fig. 1A;Supplementary Material, Supplementary Figs. S6 and 7, Supplementary Table S1). *CCNE1* amplification was found in four non-HRD cases, in-line with previous observations of almost mutual exclusivity with HRD (Goundiam *et al.*, 2015). Thus, sWGS LGAs is suitable to take over the SNP-array LSTs, which is a clinically validated method for HRD detection.

Tumor content for sWGS limits to >0.3 as estimated from the TCGA and *in silico* dilution series (Supplementary Material, Supplementary Figs. S8 and 9).

Fifteen and 20 LGAs represent soft and stringent cut-offs with sensitivity of 87.5% and 81.25% (16 cases HRD) and specificity of 90.5% and 95.2% (63 non-HRD cases), respectively, which is compatible with other state-of-the-art approaches (Fig. 1B).

To conclude, *shallowHRD* implements a fast and straightforward evaluation of tumor HRD in breast, ovarian and other cancers such as pancreatic or prostatic, performing similar to most state-of-the-art approaches, the technique is cheap and suitable for all type of samples.

## Supplementary Material

btaa261_Supplementary_Data
