# Supplementary material for: ShallowHRD: detection of homologous recombination deficiency from shallow whole genome sequencing
Source: Bioinformatics. 2020 Apr 21;36(12):3888–9. doi: 10.1093/bioinformatics/btaa261 (PMC7320600; doi:10.1093/bioinformatics/btaa261)
Supplement: btaa261_Supplementary_Data [file btaa261_supplementary_data.pdf]

Supplementary Data

ShallowHRD: Detection of Homologous Recombination Deficiency from shallow Whole Genome Sequencing (A. Eeckhoutte et al.)

Supplementary Methods 2

In-house Whole Genome Sequencing (sWGS) 2

WGS from the TCGA 2

Configuration file Control-FREEC 2

Quality control 3

HRD annotation 3

Soft and stringent HRD cut-offs and borderline HRD 3

Estimation of tumor content in WGS 4

In silico dilution series based on sWGS 4

Supplementary References 5

Supplementary Figures 6

Figure S1. Distribution of pairwise differences between segment medians in CNA profile 6

Figure S2. Two parameters characterizing quality of CNA profile 7

Figure S3. Reports of shallowHRD 9

Figure S4. Consistency in the large CNA segments in sWGS and in SNP-arrays 10

Figure S5. FFPE profiles from sWGS analyzed by shallowHRD 11

Figure S6. Large-scale CNA correspondence between sWGS and SNP-arrays 12

Figure S7. Tumor with high number of copy-neutral LOH 13

Figure S8. Example of *in silico* dilution series 14

Figure S9. Tumor content and performance of shallowHRD 16

Supplementary Table S1. Validation cohort of down-sampled WGS from the TCGA 17

Supplementary Methods

In-house Whole Genome Sequencing (sWGS)

DNA was extracted from frozen blocs (26 primary tumors, 39 Patient-Derived Xenografts, PDX) and Fixed-Formalin Paraffin Embedded (FFPE) tissues (4 primary tumors) and was sequenced on HiSeq2500 or NovaSeq (Illumina; 100bp paired-end library; coverage 0.06-1.65X; 4-6X for FFPE) and aligned on hg19 and hg38 by BWA-MEM (v0.7.15) (Li and Durbin, 2009); PDX were purified from mouse reads using XenofilteR (Kluin, et al., 2018). Optical/PCR duplicates were filtered by PicardTools (v1.140) (http://broadinstitute.github.io/picard/) and supplementary alignments were removed by Samtools (v1.9) (Li, et al., 2009).

WGS from the TCGA

WGS from the breast cancer TCGA-BRCA cohort (Weinstein, et al., 2013) (108 normal tissues, 79 primary tumors) were down-sampled to 1X by Sambamba (v0.5.9) (Tarasov, et al., 2015) on the Cancer Genomics Cloud of SevenBridges (Lau, et al., 2017).

Configuration file Control-FREEC

```
[general]

ploidy = 2,4
window = 40000
step = 20000

breakPointThreshold = 0.65
breakPointType = 2
forceGCcontentNormalisation = 1

uniqueMatch = FALSE
contaminationAdjustment = TRUE

samtools = /path/to/samtools

chrFiles = /path/to/chromFa/
chrLenFile = /path/to/hg19.len
gemMappabilityFile = /path/to/out100m2_hg19.gem

outputDir = /path/to/outputDir

BedGraphOutput = FALSE

[sample]

mateFile = /path/to/file.bam
inputFormat = BAM
matesOrientation = FR
```

Quality control

GC-corrected and normalized read counts profiles of sWGS and their sensitive segmentations (number of segments 300-1600), were annotated manually as "good" (n=55), "average" (n=6) or "bad" (n=8). Based on this annotation quality thresholds were defined.

Bad quality cases represented mainly sequencing failure independent of coverage, with frequent (n=5) poorly detectable local minimum in *M*, separating fluctuations of segments with equal copy numbers from one copy difference. Average quality was mainly due to a low coverage (0.06-0.3X) displaying high fluctuations in the number of reads per window characterized by *cMAD*. *cMAD*>0.14 and *M*>0.45 indicate low quality samples (Fig. S2). Coverage 0.3X is a low limit for sWGS to ensure prominent CNA profile.

After evaluating 108 down-sampled WGS of normal samples, a lower boundary for *CNA cut-off* was set to 0.025, to avoid CNA detection in normal and over-segmentation in low tumor content samples.

HRD annotation

In-house cases: In-house tumor cases were partially tested on the Institute Curie platform.

TCGA cohort: HRD annotation of the TCGA cohort was previously described (Manie, et al., 2016). Briefly, mutations in *BRCA1/2, RAD51C* and *PALB2* genes were searched in whole exome sequencing (WES) data; gene inactivation was considered proven when deleterious mutation and LOH (Loss Of Heterozygosity) were observed at the gene locus or two deleterious mutations found in the gene; missense mutations annotated as pathogenic in COSMIC database were considered deleterious. *BRCA1* and *RAD51C* promoter methylation was checked using the gene expression; cases with outlier low expression were annotated as HRD due to promoter methylation.

Specificity of HRD calls in SNP-array LST and scarHRD:

LST was validated on the TCGA cohort, which at the time of publication (Manie, et al., 2016) was not completely available for direct search and verification of the reported mutations. This explains relatively low specificity of LST method shown in Fig.1B. In the current validation set of the TCGA down-sampled WGS, specificity of LST method was very close to LGA in sWGS (predictions of SNP-array based method are indicated by colors in Fig.1A).

For scarHRD (Sztupinszki, et al., 2018), the methylation of *RAD51C* promoter was not assessed, which might led to missing HRD cases.

Soft and stringent HRD cut-offs and borderline HRD

Two cut-offs, soft and stringent, were introduced on the LGA number to call HRD or nonHRD. The reason for this is the appearance of HRD in breast and ovarian tumors: while the majority of cases with BRCAness (HRD) have LGA number far higher than 20, small proportion of mainly *BRCA2* mutated tumors display near-diploid genome with ~15 large-scale chromosomal breaks. From the other hand, nonHRD tumors with near-tetraploid genomes can display 15-20 large-scale chromosomal breaks. When the tumor ploidy is known, there is no problem to distinguish these two situations.

For sWGS, ploidy estimation is problematic and could introduce additional uncertainty. To overcome this issue and to bring additional attention to the low confidence of the call we introduced borderline HRD.

The Supplementary Table S1 recapitulate all the TCGA down-sampled WGS cases processed including their ID, HRD diagnostic with shallowHRD and SNP-arrays, automatic quality detection and correspondence of large segment between sWGS and SNP-array. Cases with contradictory calls are commented.

Estimation of tumor content in WGS

We used estimation of tumor content inferred from the SNP-arrays by GAP method (Popova, et al., 2009) and ichorCNA (Adalsteinsson, et al., 2017) to directly estimate tumor content in sWGS and in the dilution series using window of 50kb on all autosomal chromosomes.

In silico dilution series based on sWGS

To obtain tumor content limitation for *shallowHRD* we performed *in silico* dilution of 7 in-house sWGS by 1 sWGS with quasi-normal genome (Supplementary Figure S8A). These 8 cases were sequenced in the same batch. The dilution series was done using picardTools MergeSam (**http://broadinstitute.github.io/picard/**), recursively merging seven times the BAM file of the tumor with "quasi-normal" profile with the BAM files of other cases. The effect of the dilution is shown in Supplementary Figs. S8 B, C and D.

Three chromosomes which carried some CNA in the "quasi-normal" profile (chromosomes 3, 5 and 17) after controlFREEC processing were masked for CNA cut-off determination and LGA counting. The number of LGAs according to those dilutions is represented in Supplementary Figure S9B. *shallowHRD* presents relatively stable results with mild variation in LGA counts even for high number of sequential dilutions in good quality cases.

The tumor content estimation was based on the initial tumor content from SNP-array and calculated as proportion of mapped reads in the undiluted sWGS and the diluter sWGS. Estimations of tumor content inferred with ichorCNA (designed for cfDNA) were taken for comparison.

Even though sWGS show stable results around very low tumor content (~0.1), 0.3 could be considered as a good limit for the method application. Tumor cellularity is not directly assessed in *shallowHRD*, but rather taken into account to some extent in the automatic quality control procedure.

Supplementary References

Adalsteinsson, V.A., *et al.* (2017) Scalable whole-exome sequencing of cell-free DNA reveals high concordance with metastatic tumors, *Nat Commun*, **8**, 1324.

Chin, S.F., *et al.* (2018) Shallow whole genome sequencing for robust copy number profiling of formalin-fixed paraffin-embedded breast cancers, *Exp Mol Pathol*, **104**, 161-169.

Kluin, R.J.C., *et al.* (2018) XenofilteR: computational deconvolution of mouse and human reads in tumor xenograft sequence data, *BMC Bioinformatics*, **19**, 366.

Lau, J.W., *et al.* (2017) The Cancer Genomics Cloud: Collaborative, Reproducible, and Democratized-A New Paradigm in Large-Scale Computational Research, *Cancer Res.*, **77**, e3-e6.

Li, H. and Durbin, R. (2009) Fast and accurate short read alignment with Burrows-Wheeler transform, *Bioinformatics*, **25**, 1754-1760.

Li, H., *et al.* (2009) The Sequence Alignment/Map format and SAMtools, *Bioinformatics*, **25**, 2078-2079.

Manie, E., *et al.* (2016) Genomic hallmarks of homologous recombination deficiency in invasive breast carcinomas, *Int. J. Cancer*, **138**, 891-900.

Popova, T., *et al.* (2009) Genome Alteration Print (GAP): a tool to visualize and mine complex cancer genomic profiles obtained by SNP arrays, *Genome Biol*, **10**, R128.

Robbe, P., *et al.* (2018) Clinical whole-genome sequencing from routine formalin-fixed, paraffin-embedded specimens: pilot study for the 100,000 Genomes Project, *Genet Med*, **20**, 1196-1205.

Scheinin, I., *et al.* (2014) DNA copy number analysis of fresh and formalin-fixed specimens by shallow whole-genome sequencing with identification and exclusion of problematic regions in the genome assembly, *Genome Res.*, **24**, 2022-2032.

Sztupinszki, Z., *et al.* (2018) Migrating the SNP array-based homologous recombination deficiency measures to next generation sequencing data of breast cancer, *NPJ Breast Cancer*, **4**, 16.

Tarasov, A., *et al.* (2015) Sambamba: fast processing of NGS alignment formats, *Bioinformatics*, **31**, 2032-2034.

Van Roy, N., *et al.* (2017) Shallow Whole Genome Sequencing on Circulating Cell-Free DNA Allows Reliable Noninvasive Copy-Number Profiling in Neuroblastoma Patients, *Clin Cancer Res*, **23**, 6305-6314.

Weinstein, J.N., *et al.* (2013) The Cancer Genome Atlas Pan-Cancer analysis project, *Nat Genet*, **45**, 1113-1120.

Supplementary Figures

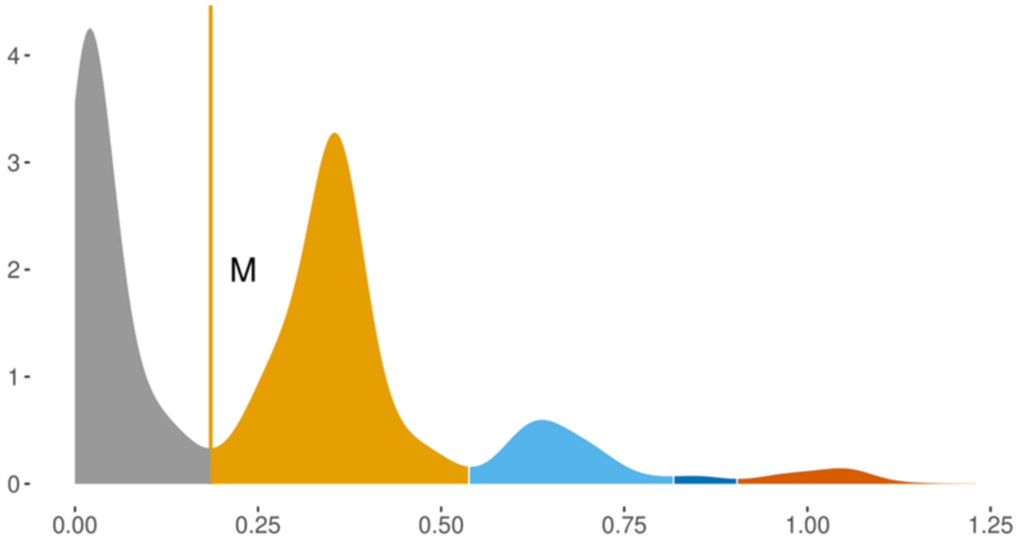

Figure S1. Distribution of pairwise differences between segment medians in CNA profile

An example of density plot of pairwise differences between segment medians is shown (only segments > 3Mb were considered). The first (grey) pick corresponds to fluctuations in segment medians related to the same copy number; the second (yellow) pick corresponds to fluctuations around the one copy difference; the third (light-blue) pick corresponds to the difference in two copies, etc. The first minimum *M* is detected (yellow vertical line). Here *M* corresponds to *CNA cut-off* used to optimize copy number segmentation and define genomic alterations. A prominent *M* evidences high signal to noise ratio in CNA profile and pure copy number states (without sub-clones).

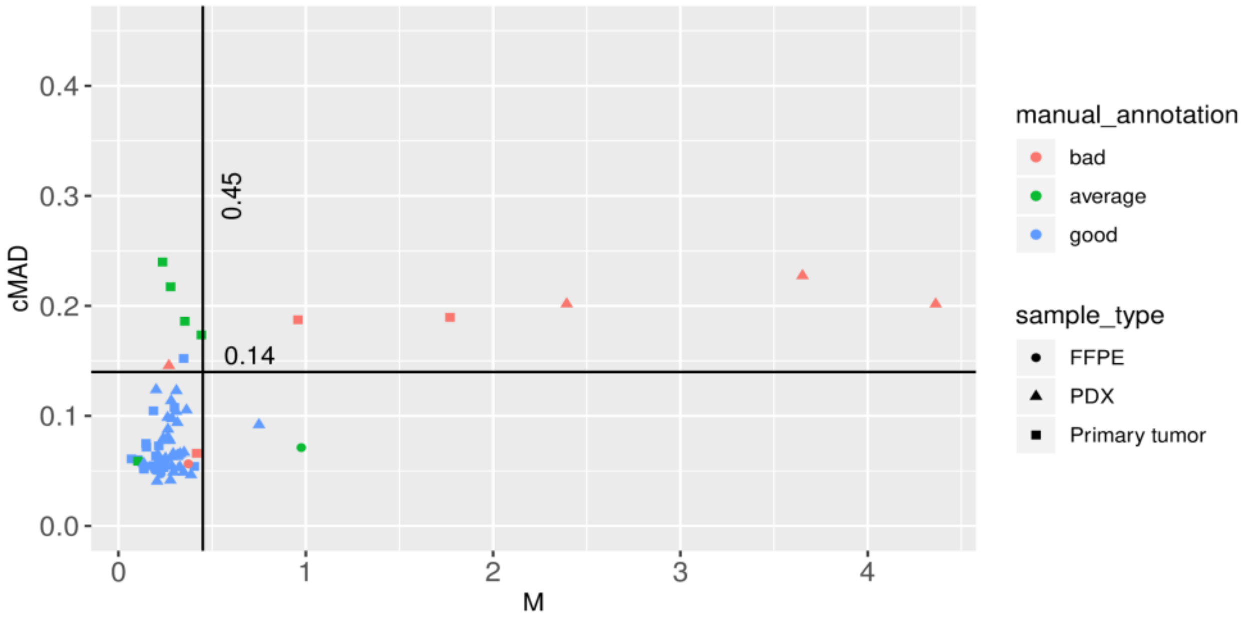

Figure S2. Two parameters characterizing quality of CNA profile

In-house sWGS CNA profiles (69 cases) manually annotated as of “bad”, “average” or “good” quality were characterized by 2 parameters: *M* defining *CNA cut-off*, and *cMAD* characterizing intra-segmental variation. These two parameters could be considered as sWGS quality markers. Two thresholds were defined: *cMAD*=0.14 and *M*=0.45 for automatic attribution of sample quality.

Bad quality cases represented mainly sequencing failure independent of coverage, with frequent (n=5) poorly detectable local minimum in *M*, separating fluctuations of segments with equal copy numbers from one copy difference. Average quality was mainly due to a low coverage (0.06-0.3X) displaying high fluctuations in the number of reads per window characterized by *cMAD*.

FFPE samples were among “good” (n=3) and “average” (n=1) quality regarding the thresholds, while two cases were actually annotated manually as “average” and “bad” (the latter due to low tumor content) (see Fig.S5 for details).

I.

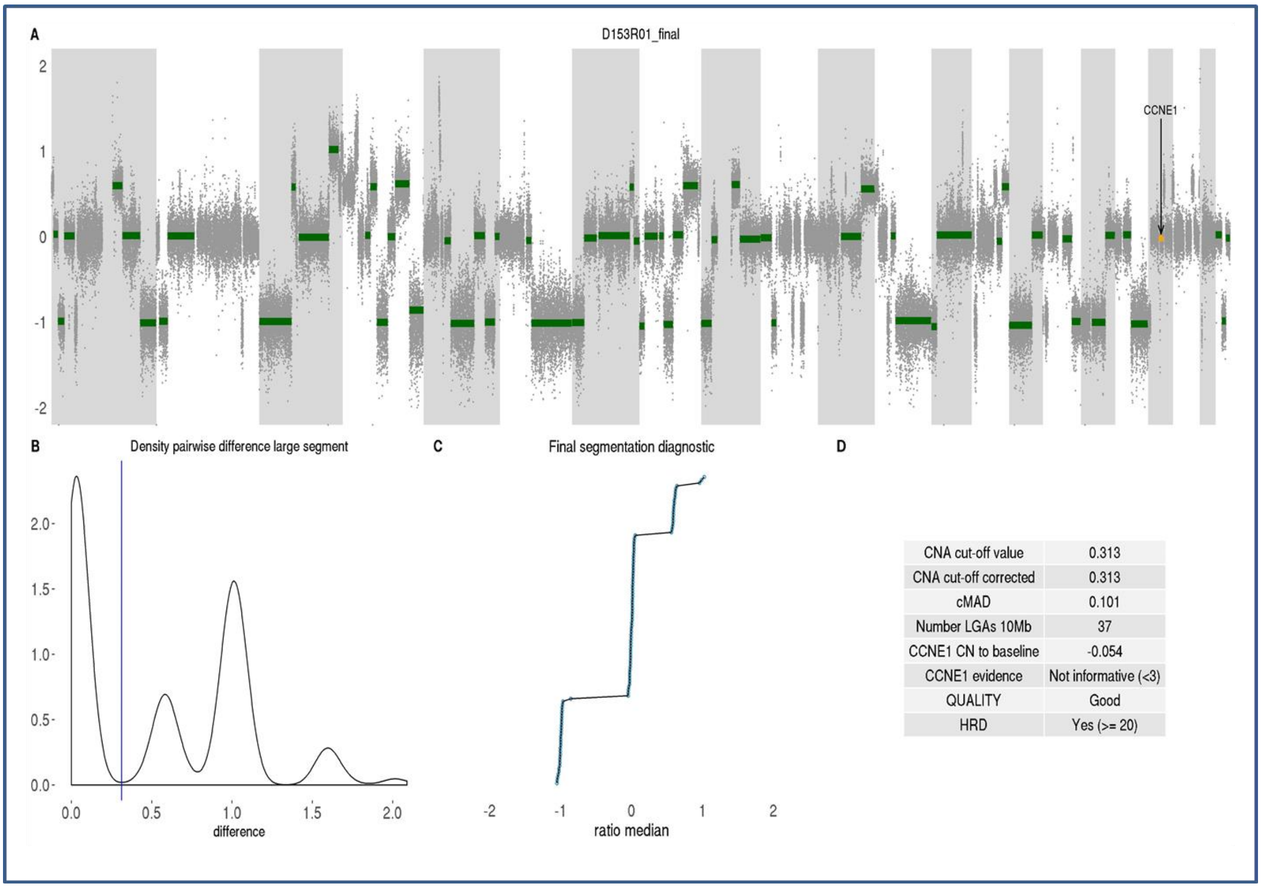
A
D153R01_final
CCNE1
B
Density pairwise difference large segment
difference
C
Final segmentation diagnostic
ratio median
D
CNA cut-off value 0.313
CNA cut-off corrected 0.313
cMAD 0.101
Number LGAs 10Mb 37
CCNE1 CN to baseline -0.054
CCNE1 evidence Not informative (<3)
QUALITY Good
HRD Yes (>= 20)

II.

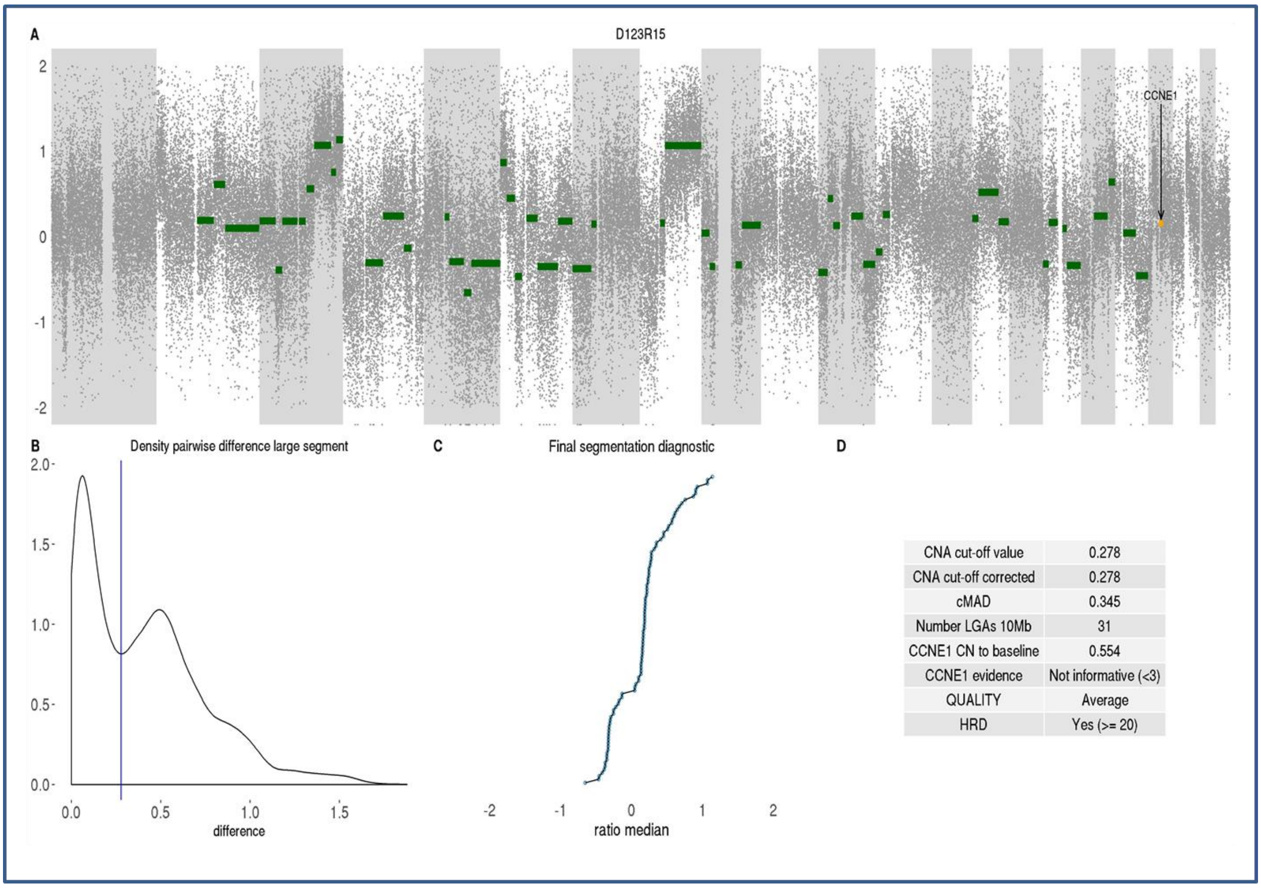
A
D123R15
CCNE1
B
Density pairwise difference large segment
difference
C
Final segmentation diagnostic
ratio median
D
CNA cut-off value 0.278
CNA cut-off corrected 0.278
cMAD 0.345
Number LGAs 10Mb 31
CCNE1 CN to baseline 0.554
CCNE1 evidence Not informative (<3)
QUALITY Average
HRD Yes (>= 20)

Figure S3. Reports of shallowHRD

I. PDX of breast cancer with *BRCA1* germline mutation; II. Primary ovarian tumor with unknown status of *BRCA1/2*

shallowHDR report contains the following items:

A. Tumor genomic profile with LGAs indicated in green.

B. Density plot of pairwise differences between large segments used to define the *CNA cut-off*.

C. Visual representation of the final segmentation, where segment medians were ordered and represented by the dots. Clear stepwise profile evidences high signal to noise ratio and proper segmentation (good quality, panel I); fuzzy profile with blurred steps evidences high unspecific variation in CNA medians with ambiguous copy number levels (average or poor quality, panel II).

D. Quality and Homologous Recombination Deficiency diagnostics including *M*, *cMAD* and LGA number with HRD status.

A.

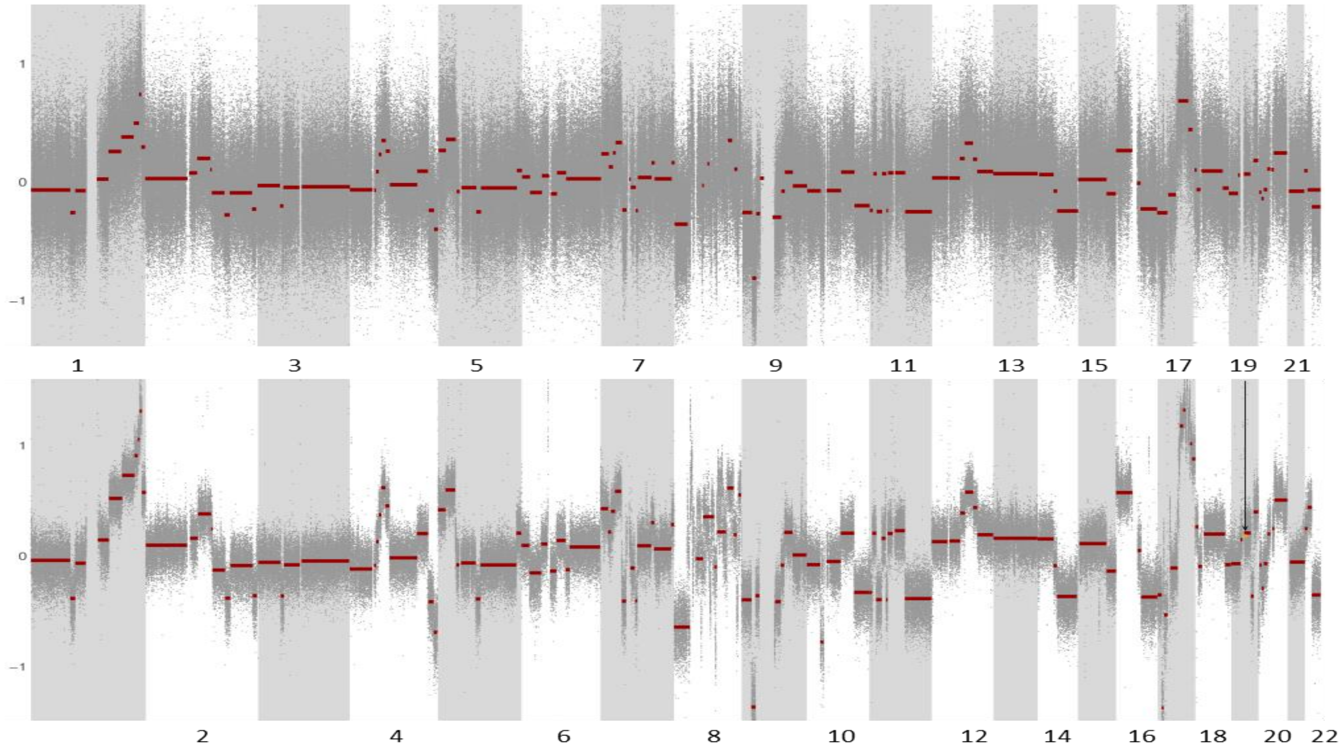

B.

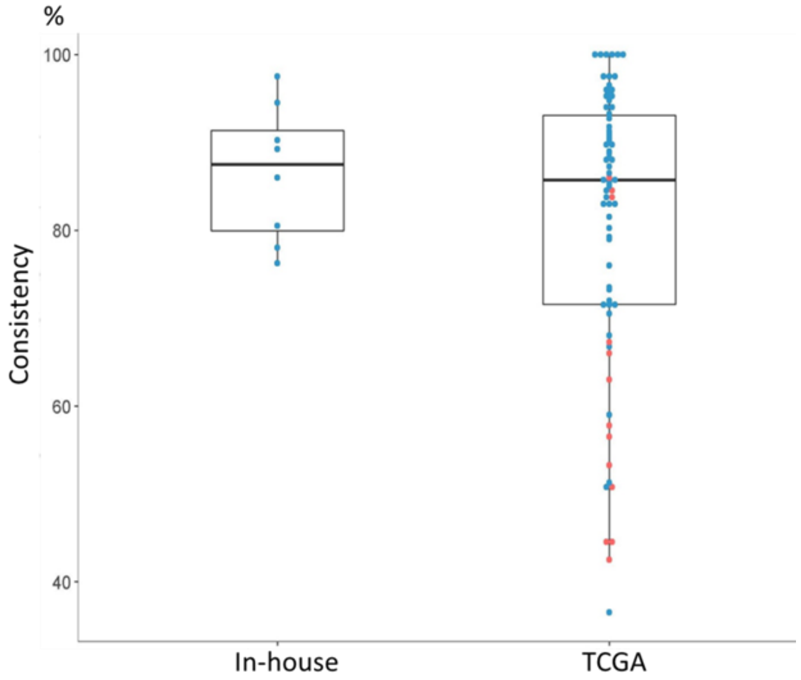

Figure S4. Consistency in the large CNA segments in sWGS and in SNP-arrays

A. Segmented CNA profiles on SNP-array (upper panel) and sWGS (lower panel) of the in-house tumor sample. Segmentation for SNP-array was optimized to absolute copy numbers using GAP method (Popova, et al., 2009) and sWGS profile was optimized by *shallowHRD* using *CNA cut-off*. Segments were considered consistent if they were both ≥10Mb in size and their boundaries were within 3Mb. sWGS CNA profile reproduced 86% of the large segments detected by SNP-arrays.
B. Overall large segments consistency (estimated as described in Figure S4A) in 8 in-house cases and 79 TCGA down-sampled WGS processed by *shallowHRD* and SNP-arrays. Red dots are cases automatically detected of average quality by *shallowHRD*.

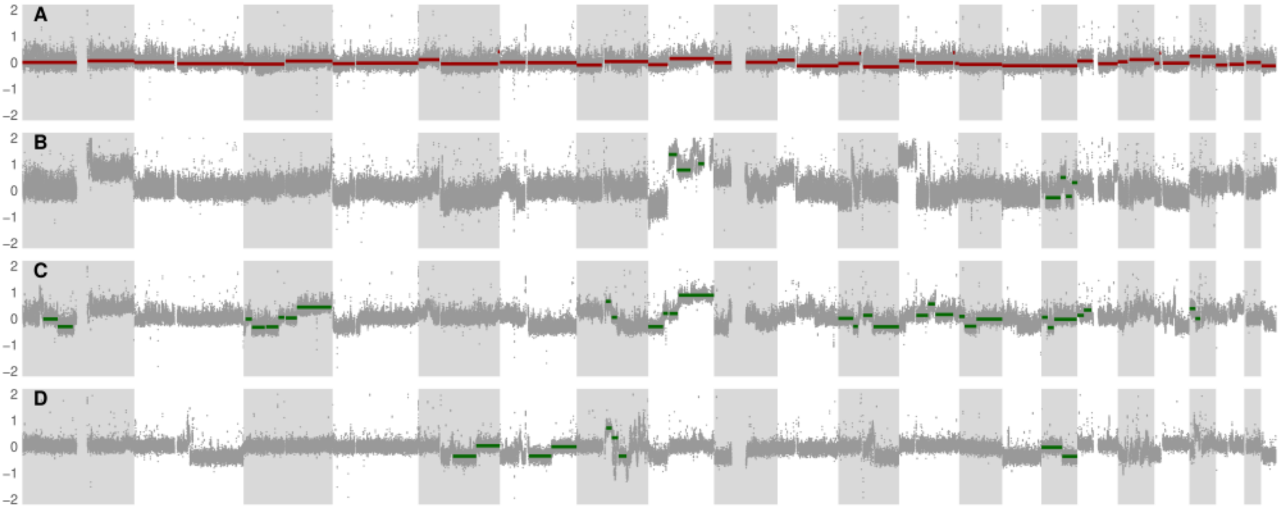

Figure S5. FFPE profiles from sWGS analyzed by shallowHRD

sWGS profiles of four FFPE cases analyzed by *shallowHRD* are shown. Segments in green correspond to LGA. The entire segmentation is indicated in red for the profile A because no LGA was detected. Profiles A, C and D are detected as “good” quality while the profile B is detected as “average” quality. Manual annotation classified sWGS of profile A as “bad” because of a low tumor content and profile B as “average. Samples B and C were correctly predicted as nonHRD and HRD (BRCA2-/-), respectively. Sample D with unknown status was predicted as nonHRD.

Overall, the limited number of cases does not allow us drive definitive conclusion but support that sWGS and therefore *shallowHRD* is applicable for FFPE cases. Moreover, several studies, including a pilot study for the 100,000 Genomes Project, investigated the use of FFPE samples for sWGS and presented good results for FFPE with WGS and CNAs interpretation (Chin, et al., 2018 ; Robbe, et al., 2018; Scheinin, et al., 2014).

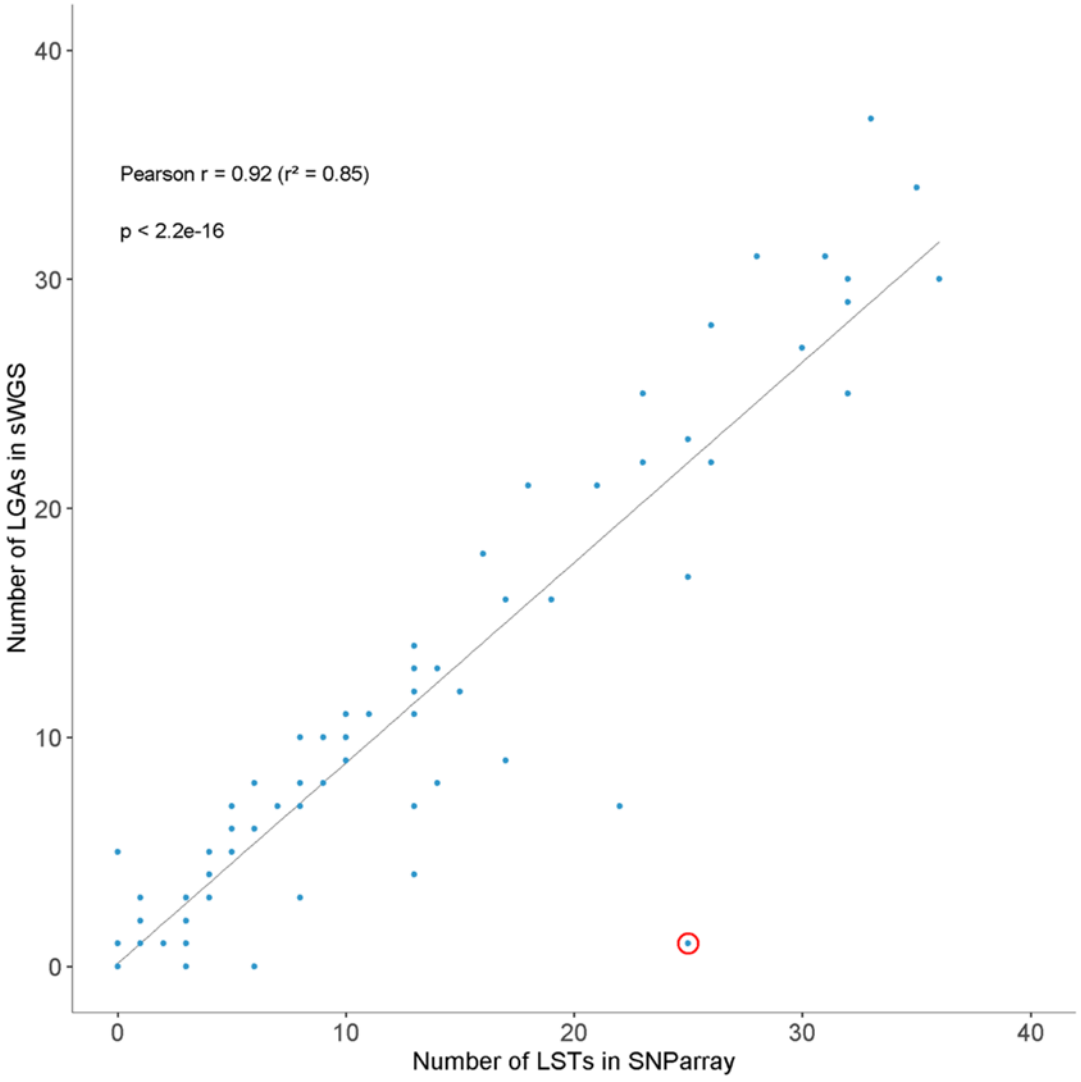

Figure S6. Large-scale CNA correspondence between sWGS and SNP-arrays

Number of LGAs in down-sampled WGS versus the number of LSTs in SNP-arrays is shown for 79 TCGA cases. The most discordant case, circled in red, is characterized by a high number of copy-neutral Loss Of Heterozygosity (see Fig. S9). Detailed information is summarized in Supplementary table S1.

A.

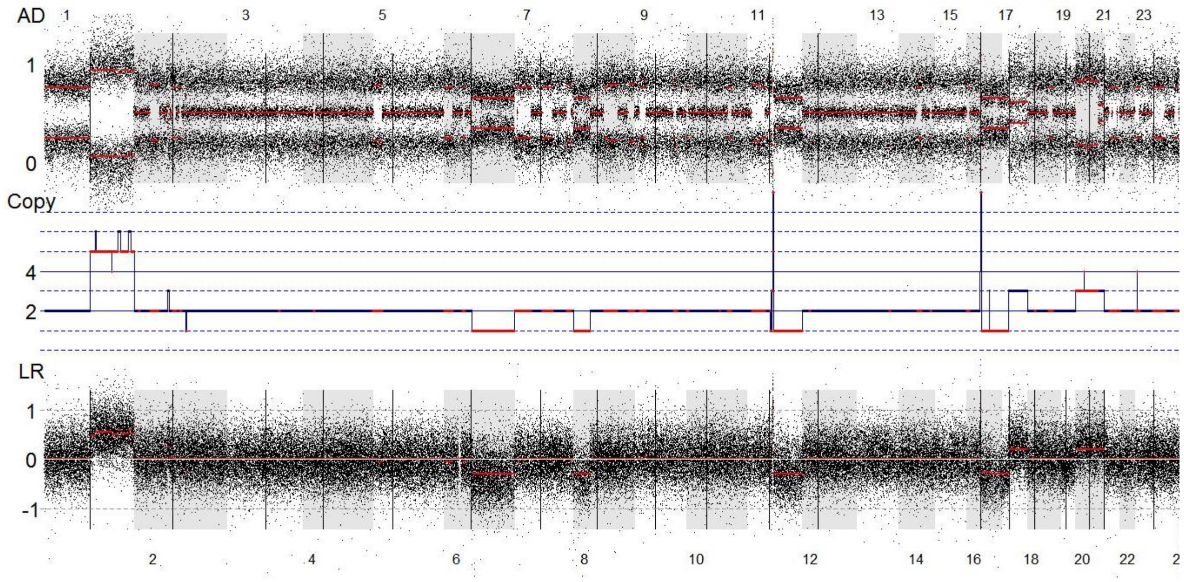

B.

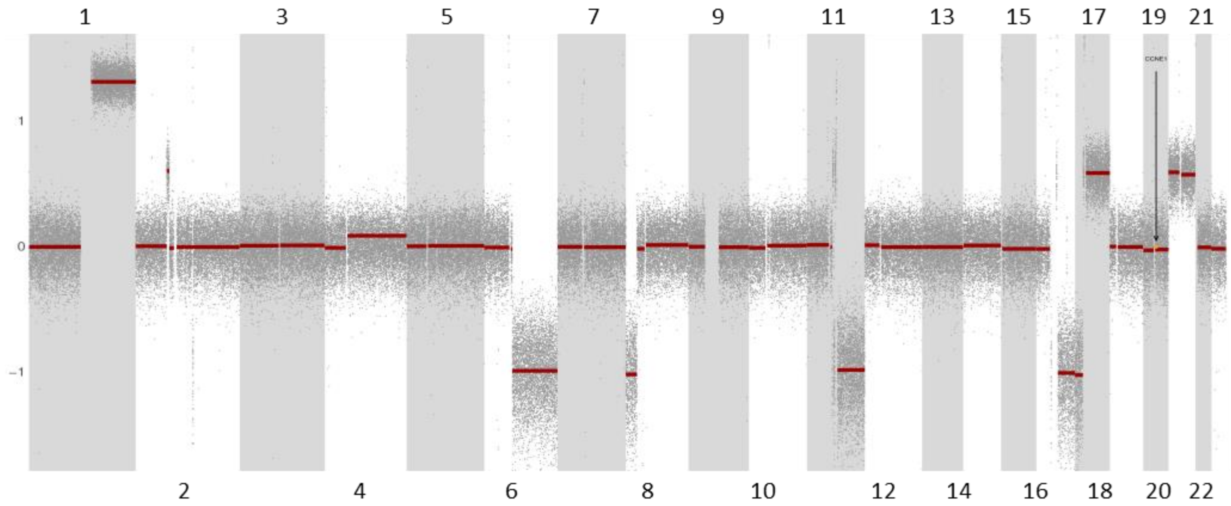

Figure S7. Tumor with high number of copy-neutral LOH

A. SNP-array copy number profile mined by GAP (Popova, et al., 2009) with numerous large-scale breakpoints detected due to copy-neutral Loss Of Heterozygosity (LOH) (ID: TCGA-EW-A1J5-01A). Top panel represents B-Allele Frequency; bottom panel represents Log ratio related to copy number alteration profile and absolute copy numbers detected by GAP software in the middle (red segments correspond to LOH). **B**. Down-sampled WGS profile of the same tumor analyzed by *shallowHRD* with a few copy number breakpoints recognized, which leaded to nonHRD prediction.

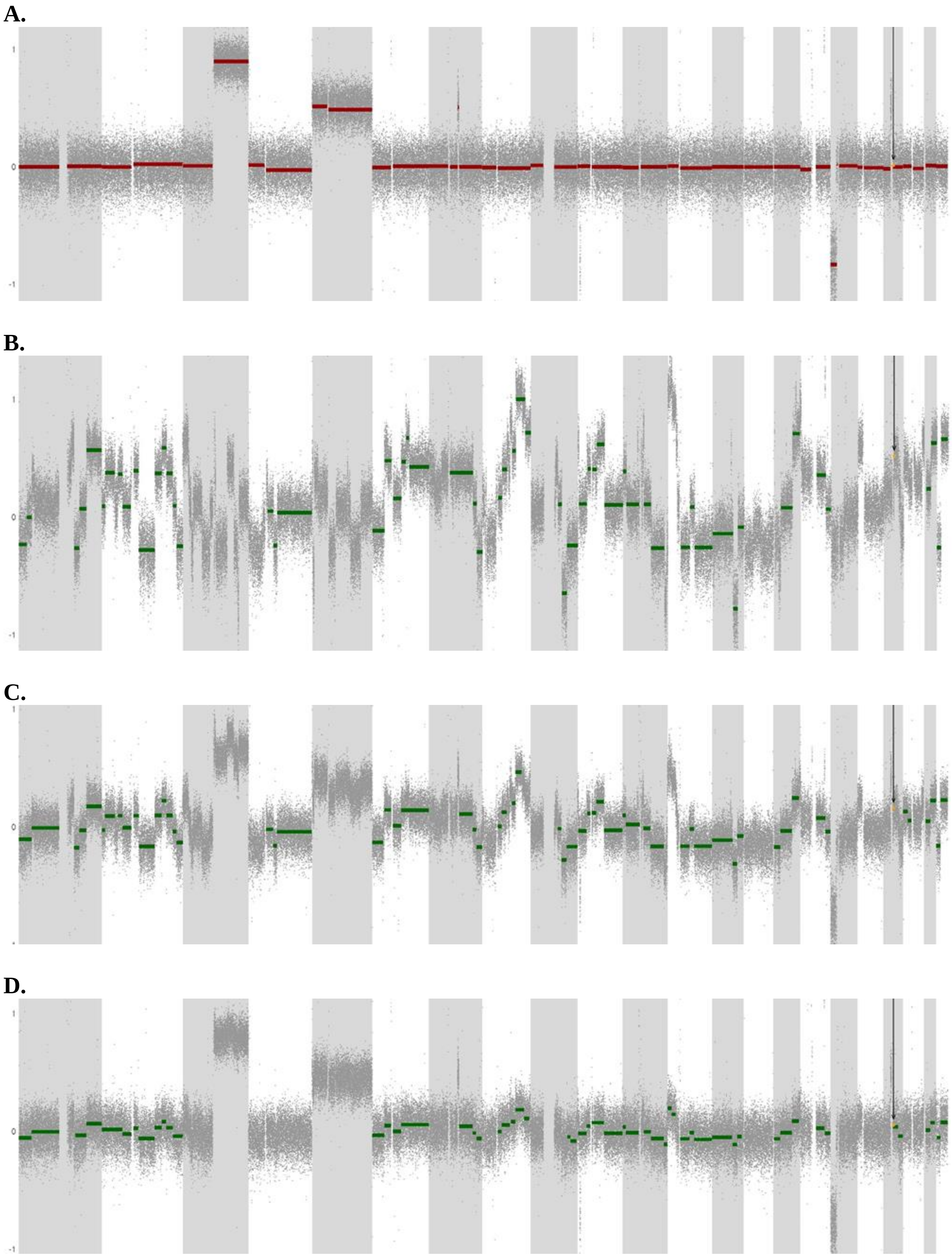

Figure S8. Example of *in silico* dilution series

Tumor with almost flat CNA profile (A), tumor with HRD (B) and *in silico* dilutions (C, D) of the tumor with HRD by the tumor with flat profile used as a “quasi-normal” counterpart.

A. In-house tumor used to make serial dilutions considered as a “quasi-normal” case. The chromosomes 3, 5 and 17 bear CNAs in this tumor and were masked from the analysis by *shallowHRD*, as detailed in Supplementary Notes. No LGA was found in this case and all segments of the final segmentation after *shallowHRD* processing are represented with red lines.

B. The tumor with HRD (shown in Supplementary Figure S6B) with SNA-array estimated tumor content 0.7. LGAs are indicated with green lines.

C. The tumor with HRD (B) diluted twice with “quasi-normal” case (A) and estimated tumor content 0.28. Tumor content was estimated regarding the initial tumor content and the proportion of reads of the tumor and “quasi-normal” sWGS.

D. The tumor with HRD (B) diluted seven times with “quasi-normal” case (A). Tumor content in this case is estimated to be 0.11.

A.

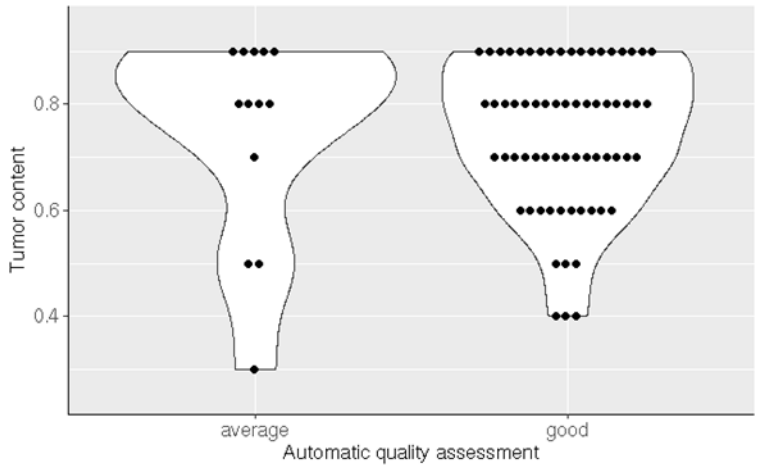

B.

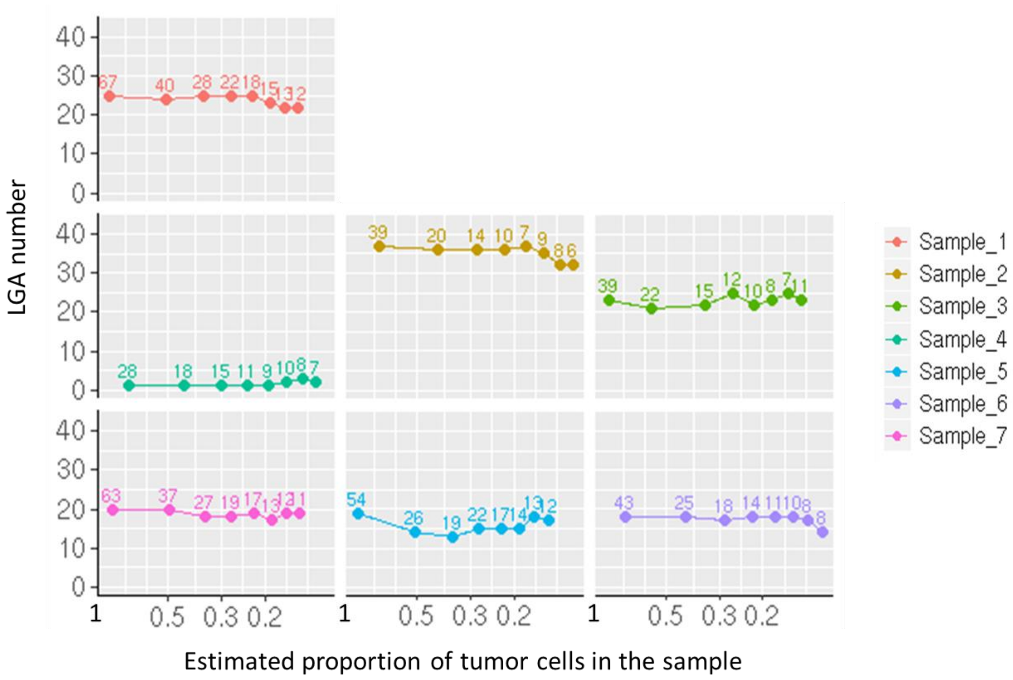

Figure S9. Tumor content and performance of shallowHRD

A. Tumor content and sample quality were shown for down-sampled WGS TCGA cases (n=79). Tumor content was taken from the corresponding SNP-arrays as estimated by GAP method (Popova, et al., 2009) and the quality assessment was automatically produced by *shallowHRD*. One case with tumor content of ~0.3 was of average quality with a low concordance between sWGS and SNP-array. Four cases of good quality had a tumor content of 0.4 and worked nicely.

B. *In silico* dilution series of in-house sWGS analyzed by *shallowHRD*. Each panel corresponds to the dilution series of one sample. Quasi-normal sample used for dilution had CNAs in chr 3, 5, 17, which were excluded from further analysis (Fig.S5A). Estimated proportion of tumor cells was shown in two ways: (1) x-axis, which is related to the tumor content estimated from the dilution (proportion of mapped reads in the undiluted sWGS and the "quasi-normal" diluter sWGS) and (2) by labels upon each point showing the percent of tumor cells evaluated directly from the diluted WGS using ichorCNA (Supplementary Methods).

Supplementary Table S1. Validation cohort of down-sampled WGS from the TCGA

TCGA ID	Tumor type	Tumor content	Proven HRD	LST diagnostic	N LST	shallowHRD diagnostic	N LGA	% large segments conserved	Detected quality	Prediction sWGS Comments
TCGA-AR-A0TU	TNBC	0.7	HRD	HRD	35	HRD	34	91	good	TP
TCGA-AO-A0J2	TNBC	0.8	HRD	HRD	32	HRD	30	73	good	TP
TCGA-A2-A04T	TNBC	0.8	HRD	HRD	31	HRD	31	79	good	TP
TCGA-A2-A3Y0	TNBC	0.7	HRD	HRD	30	HRD	27	74	good	TP
TCGA-AN-A0AT	TNBC	0.7	HRD	HRD	28	HRD	31	88	good	TP
TCGA-AN-A04D	TNBC	0.9	HRD	HRD	26	HRD	28	100	good	TP
TCGA-A7-A0CE	TNBC	0.9	HRD	HRD	25	HRD	23	91	good	TP
TCGA-AR-A256	TNBC	0.8	HRD	HRD	25	Borderline	17	51	good	Borderline<br>Low quality sWGS[1]
TCGA-AO-A124	TNBC	0.9	HRD	HRD	23	HRD	25	81	good	TP
TCGA-BH-A0WA	TNBC	0.8	HRD	HRD	23	HRD	22	67	good	TP
TCGA-EW-A1PB	TNBC	0.6	HRD	HRD	21	HRD	21	95	good	TP
TCGA-B6-A0RG	luminal	0.9	HRD	nonHRD	13	nonHRD	11	72	good	FN<br>LGA/LST consistent[2]
TCGA-AO-A0J6	TNBC	0.8	HRD	HRD	33	HRD	37	57	average	TP
TCGA-A2-A04P	TNBC	0.8	HRD	HRD	32	HRD	25	58	average	TP
TCGA-C8-A12L	TNBC	0.8	HRD	HRD	32	HRD	29	63	average	TP
TCGA-AO-A0J4	TNBC	0.7	HRD	nonHRD	17	nonHRD	9	42	average	FN<br>Low quality sWGS
TCGA-A2-A0D0	TNBC	0.8	-	HRD	36	HRD	30	83	good	FP<br>LGA/LST consistent[3]
TCGA-E2-A14P	HER2+	0.6	-	HRD	26	HRD	22	76	good	FP<br>LGA/LST consistent[3]
TCGA-EW-A1J5	luminal	0.8	-	HRD	25	nonHRD	1	93	good	TN<br>LGA/LST inconsistent (Fig S9)
TCGA-A2-A0EY	HER2+	0.7	-	nonHRD	19	Borderline	16	86	good	Borderline[4]
TCGA-E2-A1LL	TNBC	0.7	-	nonHRD	18	HRD	21	71	good	TP<br>LGA/LST inconsistent
TCGA-C8-A12Q	HER2+	0.6	-	HRD	17	Borderline	16	89	good	Borderline
TCGA-C8-A130	luminal	0.8	-	nonHRD	16	Borderline	18	36	good	Borderline<br>Low quality sWGS[1]
TCGA-B6-A0RU	TNBC	0.6	-	HRD	15	nonHRD	12	80	good	TN<br>LGA/LST inconsistent
TCGA-B6-A0RE	TNBC	0.8	-	nonHRD	14	nonHRD	13	51	good	TN
TCGA-B6-A0RE	TNBC	0.8	-	nonHRD	14	nonHRD	8	59	good	TN
TCGA-AC-A2BK	TNBC	0.9	-	nonHRD	13	nonHRD	13	86	good	TN
TCGA-AO-A0JL	TNBC	0.8	-	nonHRD	13	nonHRD	14	71	good	TN
TCGA-AO-A0JM	HER2+	0.7	-	nonHRD	13	nonHRD	12	88	good	TN
TCGA-A7-A13D	TNBC	0.7	-	nonHRD	11	nonHRD	11	85	good	TN
TCGA-BH-A1FC	TNBC	0.9	-	nonHRD	10	nonHRD	11	71	good	TN
TCGA-BH-A0H7	luminal	0.8	-	nonHRD	10	nonHRD	10	72	good	TN
TCGA-B6-A0I2	TNBC	0.6	-	nonHRD	10	nonHRD	9	96	good	TN
TCGA-BH-A18R	HER2+	0.6	-	nonHRD	10	nonHRD	11	97	good	TN
TCGA-A2-A0YG	HER2+	0.6	-	nonHRD	9	nonHRD	10	83	good	TN
TCGA-BH-A0DK	luminal	0.6	-	nonHRD	9	nonHRD	8	100	good	TN
TCGA-A8-A09X	luminal	0.4	-	nonHRD	9	nonHRD	8	94	good	TN
TCGA-E2-A15E	luminal	0.9	-	nonHRD	8	nonHRD	7	79	good	TN
TCGA-BH-A0E0	TNBC	0.7	-	nonHRD	8	nonHRD	8	94	good	TN
TCGA-BH-A0HX	luminal	0.7	-	nonHRD	8	nonHRD	8	89	good	TN

TCGA-BH-A0GY	luminal	0.6	-	nonHRD	8	nonHRD	10	90	good	TN
TCGA-A2-A04X	HER2+	0.7	-	nonHRD	7	nonHRD	7	92	good	TN
TCGA-EW-A1P8	TNBC	0.7	-	nonHRD	7	nonHRD	7	96	good	TN
TCGA-A2-A0D1	HER2+	0.9	-	nonHRD	6	nonHRD	6	87	good	TN
TCGA-BH-A0HB	luminal	0.8	-	nonHRD	6	nonHRD	8	84	good	TN
TCGA-A8-A07I	HER2+	0.9	-	nonHRD	5	nonHRD	7	85	good	TN
TCGA-E9-A1NH	luminal	0.8	-	nonHRD	5	nonHRD	5	91	good	TN
TCGA-B6-A0WX	TNBC	0.5	-	nonHRD	5	nonHRD	6	90	good	TN
TCGA-E2-A156	luminal	0.9	-	nonHRD	4	nonHRD	3	85	good	TN
TCGA-B6-A0RI	luminal	0.8	-	nonHRD	4	nonHRD	4	96	good	TN
TCGA-E2-A152	HER2+	0.8	-	nonHRD	4	nonHRD	4	83	good	TN
TCGA-A2-A3XX	TNBC	0.7	-	nonHRD	4	nonHRD	3	68	good	TN
TCGA-A7-A0D9	luminal	0.9	-	nonHRD	3	nonHRD	2	91	good	TN
TCGA-E2-A15K	luminal	0.9	-	nonHRD	3	nonHRD	2	84	good	TN
TCGA-AO-A0JJ	luminal	0.5	-	nonHRD	3	nonHRD	1	88	good	TN
TCGA-AR-A0TX	HER2+	0.4	-	nonHRD	3	nonHRD	3	93	good	TN
TCGA-BH-A0H0	luminal	0.8	-	nonHRD	2	nonHRD	1	98	good	TN
TCGA-A7-A26J	luminal	0.9	-	nonHRD	1	nonHRD	1	98	good	TN
TCGA-A7-A26J	luminal	0.9	-	nonHRD	1	nonHRD	1	86	good	TN
TCGA-B6-A0X4	luminal	0.9	-	nonHRD	1	nonHRD	1	100	good	TN
TCGA-BH-A0H6	luminal	0.9	-	nonHRD	1	nonHRD	1	71	good	TN
TCGA-A2-A259	luminal	0.7	-	nonHRD	1	nonHRD	3	90	good	TN
TCGA-E2-A15H	HER2+	0.7	-	nonHRD	1	nonHRD	1	86	good	TN
TCGA-A2-A3KC	luminal	0.6	-	nonHRD	1	nonHRD	2	100	good	TN
TCGA-AO-A0JF	luminal	0.9	-	nonHRD	0	nonHRD	5	95	good	TN
TCGA-BH-A0HK	luminal	0.9	-	nonHRD	0	nonHRD	1	97	good	TN
TCGA-AR-A2LK	luminal	0.8	-	nonHRD	0	nonHRD	0	95	good	TN
TCGA-BH-A0BM	luminal	0.7	-	nonHRD	0	nonHRD	0	100	good	TN
TCGA-BH-A0W5	luminal	0.5	-	nonHRD	0	nonHRD	0	100	good	TN
TCGA-A2-A0EU	luminal	0.4	-	nonHRD	0	nonHRD	0	95	good	TN
TCGA-BH-A0B3	TNBC	0.5	-	HRD	22	nonHRD	7	45	average	TN
TCGA-EW-A1PH	TNBC	0.9	-	nonHRD	13	nonHRD	7	53	average	TN
TCGA-A7-A26F	TNBC	0.5	-	nonHRD	13	nonHRD	4	51	average	TN
TCGA-A8-A08B	HER2+	0.9	-	nonHRD	8	nonHRD	3	66	average	TN
TCGA-A2-A04Q	TNBC	0.3	-	nonHRD	6	nonHRD	0	44	average	TN<br>Low tumor content
TCGA-E2-A109	luminal	0.9	-	nonHRD	4	nonHRD	5	67	average	TN
TCGA-A8-A092	luminal	0.9	-	nonHRD	3	nonHRD	0	84	average	TN
TCGA-A7-A0DC	NA	0.8	-	nonHRD	1	nonHRD	1	86	average	TN
TCGA-A8-A08S	HER2+	0.9	-	nonHRD	0	nonHRD	0	85	average	TN

HRD: Homologous Recombination Deficiency; LST: Large-scale State Transitions; LGA: Large Genomic Alterations; TN: true positive; TP: true negative; FN: false negative; FP: false positive.
Color code: green: no problem with the case; light orange: large sWGS segments (≥10Mb) conserved in SNP-arrays < 70%, as described in Figure S4; dark orange: “borderline” or cases with inconsistent diagnostic of HRD.

[1]: Low quality WGS due to high unspecific variation failed to be detected automatically.
[2]: BRCA2-/- can have in some rare cases low number of intra-chromosomal breaks.
[3]: Highly altered genome with no HRD evidence found (still may be HRD).
[4]: Ploidy of 4 for this case, accessible with SNParray, helping classifying the case as nonHRD.
